# Immune Cell Subtypes and Cytokines in Lung Tumor Microenvironment: Influence of COPD

**DOI:** 10.3390/cancers12051217

**Published:** 2020-05-13

**Authors:** Jun Tang, Daniel Ramis-Cabrer, Víctor Curull, Xuejie Wang, Liyun Qin, Mercé Mateu-Jiménez, Xavier Duran, Lara Pijuan, Alberto Rodríguez-Fuster, Rafael Aguiló Espases, Esther Barreiro

**Affiliations:** 1 Pulmonology Department, Muscle Wasting & Cachexia in Chronic Respiratory Diseases & Lung Cancer Research Group, Hospital del Mar-IMIM, Mar Health Park, Health and Experimental Sciences Department (CEXS), Pompeu Fabra University (UPF), Autonomous University of Barcelona (UAB), Barcelona Biomedical Research Park (PRBB), 08003 Barcelona, Spain; jun.tang2@e-campus.uab.cat (J.T.); daniel.ramis19@gmail.com (D.R.-C.); VCURULL@PARCDESALUTMAR.CAT (V.C.); Xuejie.Wang@e-campus.uab.cat (X.W.); liyun.qin@e-campus.uab.cat (L.Q.); merce.x.mateu@gsk.com (M.M.-J.); 2 Centro de Investigación en Red de Enfermedades Respiratorias (CIBERES), Instituto de Salud Carlos III (ISCIII), 08003 Barcelona, Spain; 3 Scientific, Statistics, and Technical Department, Hospital del Mar-IMIM, Mar Health Park, 08003 Barcelona, Spain; xduran@imim.es; 4 Pathology Department, Hospital del Mar-IMIM, Mar Health Park, 08003 Barcelona, Spain; LPIJUAN@PARCDESALUTMAR.CAT; 5 Thoracic Surgery Department, Hospital del Mar-IMIM, Mar Health Park, 08003 Barcelona, Spain; ARodriguezFuster@parcdesalutmar.cat (A.R.-F.); RAGUILO@PARCDESALUTMAR.CAT (R.A.E.)

**Keywords:** lung cancer, COPD, T regulatory cells, natural killer cells: immunoglobulin-secreting plasma cells, immune tumor microenvironment, IL-10 and interferon-gamma

## Abstract

Background: The immune microenvironment plays a role in tumorigenesis. Chronic Obstructive Pulmonary Disease (COPD) is an independent risk factor for lung cancer (LC). We hypothesized that immune profile characterized by T regulatory (Treg), natural killer (NK), and plasma cells, as well as interleukin (IL)-10 and interferon-gamma, may differ within tumors of LC patients with/without COPD. Methods: Treg (anti-CD3 and anti-forkhead boxP3 antibodies), NK (anti-NCR1 antibody), IgG (anti-CD138-IgG antibody), IgA (anti-CD138-IgA antibody) using immunohistochemistry, and both IL-10 and interferon-gamma (ELISA) were quantified in tumor and non-tumor specimens (thoracotomy for lung tumor resection) from 33 LC–COPD patients and 20 LC-only patients. Results: Immune profile in tumor versus non-tumor specimens: Treg cell counts significantly increased in tumors of both LC and LC–COPD patients, while in tumors of the latter group, IgG-secreting plasma cells significantly decreased and IL-10 increased. No significant differences were seen in levels of NK cells, IgA-secreting cells, IgA/IgG, or interferon-gamma. Immune profile in tumors of LC–COPD versus LC: No significant differences were observed in tumors between LC–COPD and LC patients for any study marker. Conclusions: Immune cell subtypes and cytokines are differentially expressed in lung tumors, and the presence of COPD elicited a decline in IgG-secreting plasma cell levels but not in other cell types.

## 1. Introduction

Lung cancer (LC) continues to be a major cause of mortality worldwide [1,2,3,4,5]. In certain geographical areas, LC may account for up to one-third of deaths [1,2,3,4,5,6]. The presence of airway obstruction is a major risk factor for LC development [1,2,3,4,5,6,7,8,9,10,11,12]. Specifically, Chronic Obstructive Pulmonary Disease (COPD) and emphysema [13,14,15] have been demonstrated to favor lung tumorigenesis in the patients [16,17]. The underlying biological mechanisms that render patients with COPD more susceptible to the development of emphysema remain to be fully elucidated. 

Several biological events such as increased oxidative stress, inflammation, epigenetics, and tumor microenvironment have been proposed as mechanisms that underlie the process of tumorigenesis in patients with chronic airway obstruction and emphysema [7,18]. Those events interact with key cellular mechanisms, such as angiogenesis, cell repair, and cell death and growth, which may interfere with cell survival, thus promoting tumorigenesis and LC development [7,19].

It has been well established that the tumor microenvironment and immune surveillance play a significant role in cancer initiation and progression [20,21]. Regulatory T cells (Treg) are key in immune tolerance and homeostasis [22,23]. Treg cells infiltrate tumors and suppress antitumor immunity within the tumor microenvironment, thus promoting tumor progression and growth [22,23]. Importantly, it has also been shown that tumor-infiltrating Treg cells express a differential phenotype from that expressed in circulating cells [24,25], which implies that local environmental factors may influence the immunosuppressive function of Treg cells. Whether chronic airway obstruction, such as in COPD, may alter Treg expression remains to be investigated.

Natural killer (NK) cells, which are present in peripheral blood, lymph nodes, spleen, and bone marrow, play important roles in innate and adaptive immune system responses [26,27]. NK cells activate monocytes and cytotoxic T cells and modulate T helper cell polarization, while they may also stimulate or inhibit B cells to produce immunoglobulins [28]. NK cells also release cytokines such as interferon-gamma that inhibit the proliferation of lung tumors [29]. Moreover, tumor cells may also produce immunosuppressive cytokines, namely interleukin (IL)-10 and transforming growth factor (TGF) beta that inhibit the function of NK cells [30,31,32,33,34]. Whether the presence of COPD may modify NK cell counts in tumors remains to be explored. Tumor-infiltrating B cells and antibodies produced within the tumors may also play a role in cancer progression. Furthermore, high levels of IgG and low levels of IgA within lung tumors were associated with better overall survival for certain adenocarcinoma subtypes [35]. Whether the presence of airway obstruction may influence the expression of plasma cells remains unanswered. 

On this basis, we hypothesized that, in LC patients with COPD, the immune profile characterized by the expression of Treg cells, NK cells, plasmatic cells, and levels of the cytokines’ interferon-gamma and IL-10 within the tumors may differ from LC patients with no underlying COPD. Accordingly, our objectives were to determine in lung tumors and non-tumor specimens of LC patients, with and without COPD, the following parameters: (1) counts of Treg and NK cells, (2) numbers of both IgG- and IGA-secreting plasma cells, and (3) levels of the cytokines IL-10 and interferon-gamma.

## 2. Methods

### 2.1. Study Design and Ethics

This is a cross-sectional prospective study designed by following the World Medical Association guidelines (Seventh revision of the Declaration of Helsinki, Fortaleza, Brazil, 2013) [36] for research on human beings and approved by the institutional Ethics Committee on Human Investigation (protocol # 2008/3390/I, Hospital del Mar–IMIM, Barcelona, Spain). All patients invited to participate in the study signed the informed written consent.

Patients were prospectively recruited from the Lung Cancer Clinic of the Respiratory Medicine Department at *Hospital del Mar* (Barcelona, Spain). All the patients were part of the *Lung Cancer Mar Cohort*. For this observational study, 53 patients with LC were recruited during the years 2017–2019. Candidates for tumor resection underwent pulmonary surgery prior to administration of any sort of adjuvant therapy. LC diagnosis and staging were established by histological confirmation and classified according to currently available guidelines for the diagnosis and management of LC [37,38]. TNM (tumor, node, and metastasis) staging was defined as stated in the eighth edition of the Lung Cancer Stage Classification [39]. COPD diagnosis was established as a post-bronchodilator forced expiratory volume in one second (FEV1)/forced vital capacity (FVC) ≤ 0.7, which is not fully reversible by spirometry, according to currently available guidelines for diagnosis and management of COPD [5,40]. Exclusion criteria were as follows: small cell lung cancer (SCLC), chronic cardiovascular disease, restrictive lung disease, metabolic, immune disease, or clot system disorders, signs of severe inflammation and/or bronchial infection (bronchoscopy), current or recent invasive mechanical ventilation, or long-term oxygen therapy.

Specimens from the tumor and non-tumor lungs were collected from all the study subjects. Patients were further subdivided post hoc into two groups on the basis of underlying COPD: (1) 33 patients with LC and COPD (LC–COPD group) and (2) 20 patients with LC without COPD (LC group). 

### 2.2. Clinical Assessment

In all patients, lung function parameters were assessed by following standard procedures. Diagnosis and severity of patients with COPD were determined according to currently available guidelines [5,40]. Nutritional evaluation included the assessment of body mass index (BMI) and nutritional blood parameters from all patients. 

### 2.3. Sample Collection and Preservation

Lung samples were obtained from tumors and the surrounding non-tumor parenchyma, following standard technical procedures during thoracotomy for the standard care in the treatment of lung tumors. In all patients, the expert pulmonary pathologist selected tumor and non-tumor lung specimens of approximately 10 × 10 mm^2^ area from the fresh samples, as previously validated [7,8,9]. Non-tumor specimens were collected as far as possible from the distal to the tumor margins (average >7 cm). Fragments of both tumor and non-tumor specimens were fixed in formalin and embedded in paraffin blocks until further use. Another fragment was snap-frozen immediately in liquid nitrogen and stored at −80 °C for the quantification of the cytokine levels. 

### 2.4. Identification of Treg Cells and Plasma Cells in the Lung Specimens

Treg cells and IgG and IgA immunoglobulins secreting plasma cells were identified on three-micrometer lung tumor and non-tumor cross-sections, using double-staining immunohistochemical procedures (EnVision DuoFLEX Doublestain System, Dako North America Inc., Carpinteria, CA, USA) following the manufacturer’s instructions and previous studies [7]. Treg cells were identified through the expression of CD3 and the intracellular transcription factor-forkhead box P3 (FOXP3), using specific antibodies (anti-CD3 and anti-FOXP3 clone 236A/E7, respectively, Dako North America and Abcam, Cambridge, UK, respectively). Plasma cells were identified by using the CD138 marker and the corresponding immunoglobulins A and G (anti-CD138 clone MI15, anti-IgA, and anti-IgG, respectively, Dako North America). Following deparaffinization, lung sample cross-sections were immersed in preheated antigen-retrieval solution (Dako high pH solution) at 95 ℃ for 20 min, to be then allowed to cool down to room temperature. Slides were washed several times with wash buffer (Dako wash buffer solution). Endogenous peroxidase activity was blocked for minutes with Dako endogenous enzyme blocking agent. Samples were incubated with the corresponding primary antibodies: anti-human CD3 rabbit polyclonal antibody or anti-human CD138 mouse monoclonal antibody for 40 min. The second incubation was performed for 1 h with the corresponding antibody in each case (anti-human FOXP3 mouse monoclonal antibody, anti-human IgA, or IgG rabbit polyclonal antibody). Chain-polymer conjugate technology utilizing enzyme-labeled inert backbone molecule of dextran was used in order to amplify the signal (EnVision DuoFLEX, Dako) [41]. Samples were then incubated with horseradish peroxidase (HRP) for mouse monoclonal antibodies and alkaline phosphatase (AP) for rabbit polyclonal antibodies for 20 minutes. Slides were gently washed and incubated for 10 min with diaminobenzidine (EnVision DuoFLEX DAB+, Carpinteria, CA, USA), as a chromogen for mouse monoclonal antibodies (brown reaction product; anti-FOXP3 or anti-CD138 antibodies) and liquid permanent red (EnVision DuoFLEX LPR) as a chromogen for rabbit polyclonal antibodies (red reaction product; anti-CD3, anti-IgA or anti-IgG antibodies). 

All procedures were conducted at room temperature. Hematoxylin counterstaining was performed for two minutes, and slides were mounted for conventional microscopy. Images were taken under a light microscope (Olympus, Series BX50F3, Olympus Optical Co., Hamburg, Germany) coupled with an image-digitizing camera (Pixera Studio, version 1.0.4, Pixera Corporation, Los Gatos, CA, USA). The number of cells and total area (μm^2^) were measured in each of the lung specimens (both tumor and non-tumor samples), using Image J software (National Institute of Health, Maryland, MD, USA).

In each lung section, the total amount of Treg cells (both CD3- and FOXP3-positively-stained), plasma cells secreting IgA (CD138- and IgA-positively-stained), and plasma cells secreting IgG (CD138- and IgG-positively stained) were quantified blindly by two independent observers who were previously trained for that purpose (correlation between them R^2^ > 0.90). In order to ensure the quality and reliability of the results, the discrepant results were measured again by the two independent observers, as many times as a correlation > 0.90 was achieved for each sample and analyzed marker. All the results are presented as follows: (1) as the percentage of Treg cells in the measured area in μm^2^ in both tumor and non-tumor lung specimens (% Treg, total number of cells/μm^2^ × 100), and (2) as the percentage of either IgA or IgG positive plasma cells in the measured area in μm^2^ in both tumor and non-tumor lung specimens (% IgA, total number of plasma cells/μm^2^ × 100 and % IgG, total number of plasma cells/μm^2^ × 100 respectively). The ratio of IgA to IgG was also calculated by dividing the % of IgA-secreting plasma cells for the given area by the % of IgG-secreting plasma cells within the same area (no units).

### 2.5. Identification of NK Cells in Lung Specimens

NK cells were identified in the tumor and non-tumor lung specimens on three-micrometer sections, using conventional immunohistochemical procedures as previously described [7]. Following deparaffinization, lung cross-sections were immersed in preheated antigen retrieval solution of ethylenediaminetetraacetic acid (EDTA, pH 8, Sigma-Aldrich, St. Louis, MO, USA), incubated at 95 ℃ for 20 min, and then cooled down to room temperature. Slides were washed over the following steps with phosphate buffer saline (PBS, Sigma-Aldrich). Endogenous peroxidase activity was blocked with 3% hydrogen peroxide for 15 min. In order to properly identify NK cells in the lung samples (tumor and non-tumor specimens), NKp46 receptor (encoded by the *ncr*1 gene) was measured by using a specific primary antibody, as also previously reported [42,43]. Thus, primary antibody incubation with anti-natural Cytotoxicity Triggering Receptor 1 (anti-NCR1 protein antibody, Abcam, Cambridge, UK) was performed for one hour. Slides were then incubated with biotinylated universal secondary antibody for 30 min, followed by another 30 min incubation with HRP-streptavidin and diaminobenzidine for five minutes (kit LSAB+HRP Dako Cytomation Inc., Carpinteria, CA, USA) as a substrate. Hematoxylin counterstaining was performed, and slides were dehydrated and mounted for conventional microscopy. Images of the stained lung sections (tumor and non-tumor) were captured with a light microscope (Olympus, Series BX50F3, Olympus Optical Co., Hamburg, Germany) coupled with an image-digitizing camera (Pixera Studio, version 1.0.4, Pixera Corporation, Los Gatos, CA, USA). In addition, NCR1-positively-stained cells were counted in the tumor and non-tumor lung specimens of all the patients. The area of the lungs in which NK cells were identified (μm^2^) was also measured in both tumor and non-tumor specimens, using Image J software (National Institutes of Health, USA). Data are shown as the percentage of NK cells in the measured area in both tumor and non-tumor lung specimens (% NK cells/μm^2^ × 100).

### 2.6. Quantification of Cytokines in Lung Tissue

Protein levels of IL-10 and interferon-gamma were quantified in tumor and non-tumor lung specimens from all the subjects, using specific Enzyme-Linked Immunosorbent Assay (ELISA) kits (Raybiotech Inc, Norcross GA), following the manufacturer’s instructions and previous studies [7]. Frozen samples from all the patients were homogenized in lysis buffer. Samples were centrifuged at 1000× *g* for 30 min, the pellet was discarded, and the supernatant was designated as the crude cytoplasmic homogenate. The entire procedures were always conducted at 5 ℃ (on ice). In the assigned ELISA plates, 100 μL of lung homogenates were added and incubated with the corresponding diluted biotinylated antibody in duplicates. After several washes with washing solution, samples were incubated with HRP, to be subsequently incubated with tetramethylbenzidine (TMB, Raybiotech Inc, Norcross, GA, USA) substrate solution at room temperature, in darkness. Finally, the enzyme reaction (HRP) was suspended by the addition of stop solution reagent to all the samples. A standard curve was always created with each assay run. Absorbance was read in a microplate reader at 450 nm, using 655 nm as a reference filter. Intra- and inter-assay coefficients of variation in lung homogenates ranged from 0.45% to 3.52% and from 0.89% to 3.69% for both IL-10 and in interferon-gamma ELISA experiments, respectively. 

### 2.7. Statistical Analyses

All the statistical analyses were performed by using STATA (software for Statistics and Data Science) software (StataCorp LLC, College Station, TX, USA). The normality of the study variables was tested by using the Shapiro–Wilk test. Clinical variables are expressed in a Table 1. Qualitative variables are represented as frequencies (number and percentage), while quantitative variables are shown as mean and standard deviations. Differences in clinical variables between LC and LC–COPD groups of patients were assessed by using the Student’s *t*-test. Histological results obtained in the lung preparations are expressed as scatter plots of individual values in which median and interquartile ranges are also shown. Differences between patient groups (LC and LC–COPD) and types of samples (tumor and non-tumor) were assessed by using the Kruskal–Wallis equality-of-populations rank test, followed by Dunn’s Pairwise Comparison test (Sidák adjustment). Statistical significance was established at *p* ≤ 0.05.

## 3. Results

### 3.1. Clinical Characteristics

Clinical and functional characteristics of LC and LC–COPD patients that were recruited in the current investigation are shown in Table 1. As expected, the number of LC–COPD patients was higher than those in the group of LC. Age did not significantly differ between the two groups of patients, while BMI was significantly lower in LC–COPD patients compared to LC patients. The number of male patients in the LC–COPD group was greater than in LC patients. As expected, current smokers and the number of pack/year was significantly greater in LC–COPD patients compared to LC patients, while the number of never smokers was significantly greater in the latter group (Table 1). The lung functional parameters FEV_1_, FEV_1_/FVC, DL_CO_, and K_CO_ in LC–COPD patients were significantly lower than in LC patients (Table 1). Most of the patients with COPD were in GOLD I and II stages (90%). TMN staging or histological subtypes did not significantly differ between the two groups. The number of patients with adjuvant treatment following thoracotomy did not differ between the two study groups. In LC–COPD compared to LC patients, the levels of total leucocytes and neutrophils were significantly increased while levels of albumin significantly decreased. Total proteins, fibrinogen, C-reactive protein (CRP), globular sedimentation velocity (GSV), and body weight loss did not differ between LC–COPD and LC patients. 

### 3.2. Treg and NK Cells in Lung Specimens

#### 3.2.1. Differences between LC–COPD and LC in either Tumor Lesions or Non-Tumor Specimens

**Figure 1:**
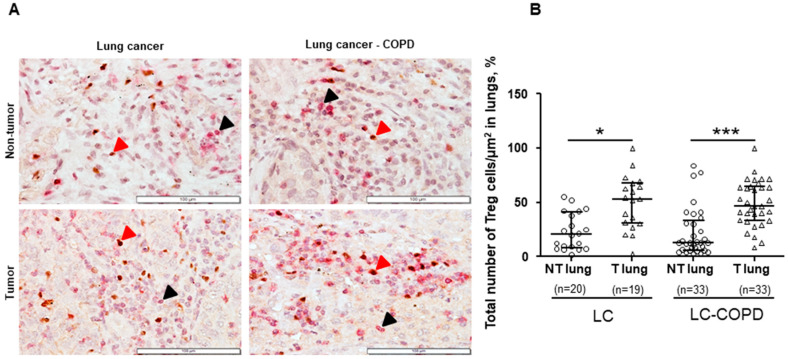
(**A**) Representative examples of double immunohistochemical staining for Treg cells (CD3-FOXP3 positively stained T cells) in LC and LC–COPD patients, respectively. All types of T cells (CD3+) are stained in only in red (black arrow), while Treg cells (CD3+-FOXP3+) are specifically stained with both brown and red. (**B**) Median and interquartile ranges between 75^th^ and 25^th^ percentiles of proportions of Treg cells in the total measured area. Comparisons were made between the non-tumor (NT) and tumor (T) samples and the LC and LC–COPD groups. For technical reasons, the number of patients in each group or type of samples (tumor and non-tumor) may differ. Statistical significance: *, *p* ≤ 0.5; ***, *p* ≤ 0.001 between tumor and non-tumor lungs in either LC or LC–COPD patients. Definition of abbreviations: LC, lung cancer; COPD, chronic obstructive pulmonary disease; Treg, regulatory T cells; CD, cluster of differentiation; FOXP3, forkhead box P3.

**Figure 2:**
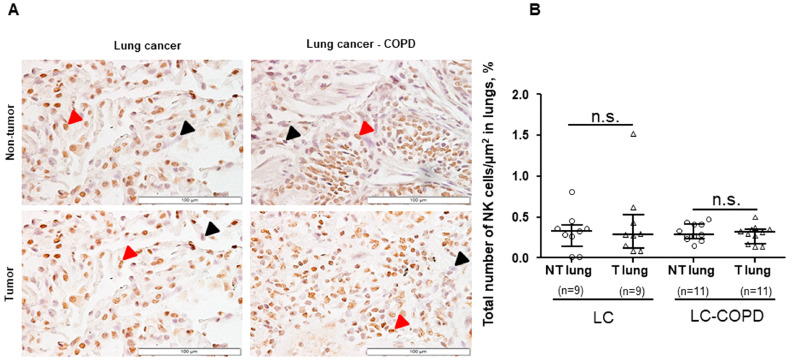
(**A**) Representative examples of double immunohistochemical staining for NK cells (NCR1+) in LC and LC–COPD patients, respectively. Black arrows point toward NK cells negatively stained in blue with hematoxylin, and red arrows point toward NK cells (NCR1+) stained in brown. (**B**) Median and interquartile ranges between 75th and 25th percentiles of proportions of NK cells in the total measured area. Comparisons were made between the non-tumor (NT) and tumor (T) samples and the LC and LC–COPD groups. For technical reasons, the number of patients in each group or type of samples (tumor and non-tumor) may differ. Statistical significance: n.s. No significance between tumor and non-tumor lungs in either LC or LC–COPD patients. Definition of abbreviations: LC, lung cancer; COPD, chronic obstructive pulmonary disease; CD, cluster of differentiation, NK, natural killer; NCR1, natural cytotoxicity triggering receptor 1.

No significant differences were found in the total proportions of Treg cells/μm^2^ or NK cells/μm^2^ between LC–COPD and LC patients in either tumor or non-tumor specimens (Figure 1 and Figure 2). A subanalysis conducted only in patients with lung adenocarcinoma revealed identical results for this set of experiments 

#### 3.2.2. Differences between Tumor and Non-Tumor Parenchyma in LC–COPD and LC Patients

Proportions of Treg cells/μm^2^ significantly increased in the tumors compared to non-tumor specimens in both LC and LC–COPD patient groups (Figure 1A,B). However, no significant differences were found in the proportions of NK cells/μm^2^ between the tumor and non-tumor lung specimens in either LC or LC–COPD patients (Figure 2A,B). A subanalysis conducted only in patients with lung adenocarcinoma revealed identical results for this set of experiments 

### 3.3. IgG and IgA Secreting Plasma Cells in Lung Specimens

#### 3.3.1. Differences between LC–COPD and LC in either Tumor Lesions or Non-Tumor Specimens

**Figure 3:**
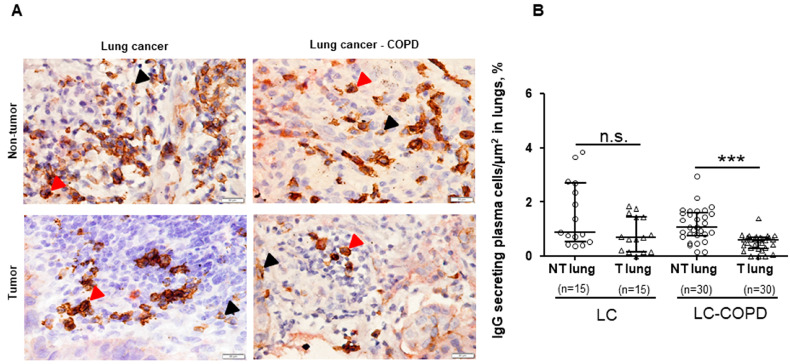
(**A**) Representative examples of double immunohistochemical staining for IgG-secreting plasma cells (CD138-IgG positively stained plasma cells) in LC and LC–COPD patients, respectively. All types of plasma cells (CD138+) are stained only in brown (black arrow), while IgG-secreting plasma cells (CD138+IgG+) are specifically stained with both brown and red. (**B**) Median and interquartile ranges between 75th and 25th percentiles of number of IgG-secreting plasma cells in the total measured area. Black-stained regions within the lungs correspond to anthracosis. Comparisons were made between the non-tumor (NT) and tumor (T) samples and the LC and LC–COPD groups. For technical reasons, the number of patients in each group or type of samples (tumor and non-tumor) may differ. Statistical significance: ***, *p* ≤ 0.001 between tumor and non-tumor lungs in LC–COPD patients. Definition of abbreviations: LC, lung cancer; COPD, chronic obstructive pulmonary disease; CD, cluster of differentiation; Ig, immunoglobulin.

**Figure 4:**
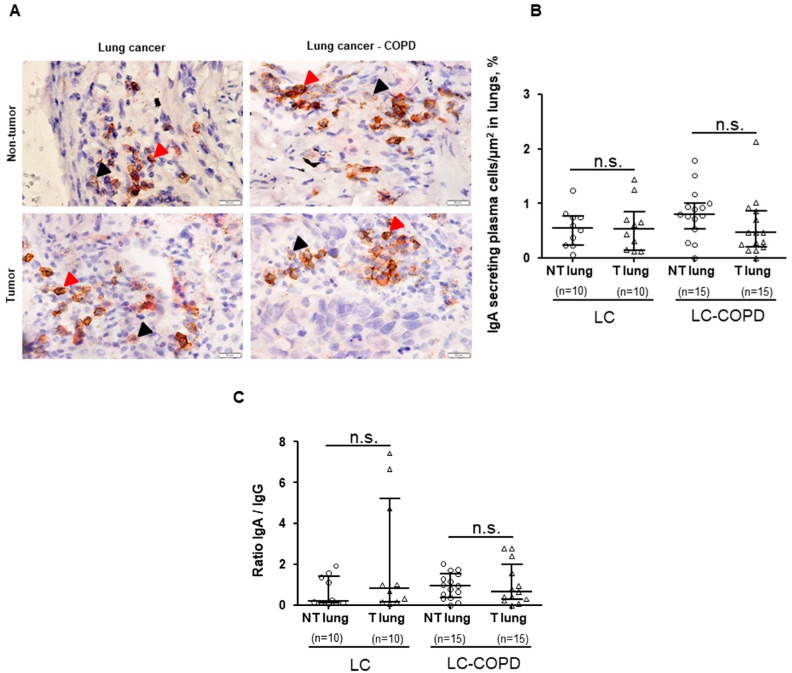
(**A**) Representative examples of double immunohistochemical staining for IgA-secreting plasma cells (CD138-IgA positively stained plasma cells) in LC and LC–COPD patients, respectively. All types of plasma cells (CD138+) are stained only in brown (black arrow), while IgA-secreting plasma cells (CD138+IgA+) are specifically stained with both brown and red. (**B**) Median and interquartile ranges between 75th and 25th percentiles of number of IgG-secreting plasma cells in the total measured area. Black-stained regions within the lungs correspond to anthracosis. (**C**) Median and interquartile ranges between 75th and 25th percentiles of IgA/IgG ratio in LC and LC–COPD patients, respectively. Comparisons were made between the non-tumor (NT) and tumor (T) samples and the LC and LC–COPD groups. For technical reasons, the number of patients in each group or type of samples (tumor and non-tumor) may differ. Statistical significance: n.s. No significance between tumor and non-tumor lungs in either LC or LC–COPD patients. Definition of abbreviations: LC, lung cancer; COPD, chronic obstructive pulmonary disease; CD, cluster of differentiation; Ig, immunoglobulin.

No significant differences were found in the total proportions of IgG-secreting plasma cells/μm^2^ or IgA-secreting plasma cells/μm^2^ between LC–COPD and LC patients, in either tumor or non-tumor specimens (Figure 3 and Figure 4). A subanalysis conducted only in patients with lung adenocarcinoma revealed identical results for this set of experiments 

#### 3.3.2. Differences between Tumor and Non-Tumor Parenchyma in LC–COPD and LC Patients

Proportions of IgG-secreting plasma cells/μm^2^ significantly decreased in the tumors compared to non-tumor specimens, only in LC–COPD patients (Figure 3A,B). No significant differences were found in the proportions of IgA-secreting plasma cells/μm^2^ between tumor and non-tumor lung specimens in either LC or LC–COPD patients (Figure 4A,B). No significant differences were found in the IgA/IgG ratio between the tumor and non-tumor lung specimens in either LC or LC–COPD patients (Figure 4C). A subanalysis conducted only in patients with lung adenocarcinoma revealed identical results for this set of experiments 

### 3.4. Cytokines Levels in Lung Specimens

#### 3.4.1. Differences between LC–COPD and LC in either Tumor Lesions or Non-Tumor Specimens

**Figure 5:**
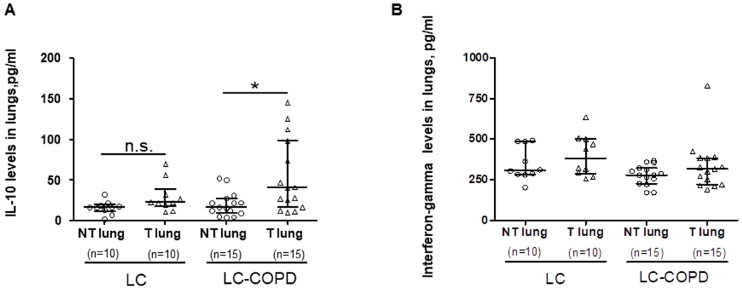
(**A**) Mean values and SD of number IL-10 levels by ELISA in LC and LC–COPD patients, respectively. (**B**) Median and interquartile ranges between 75th and 25th percentiles of number of interferon-gamma levels by ELISA in LC and LC–COPD patients, respectively. Comparisons were made between the non-tumor (NT) and tumor (T) samples and the LC and LC–COPD groups. For technical reasons, the number of patients in each group or type of samples (tumor and non-tumor) may differ. Statistical significance: *, *p* ≤ 0.5 between tumor (T) and non-tumor (NT) lungs in LC–COPD patients. Definition of abbreviations: LC, lung cancer; COPD, chronic obstructive pulmonary disease; IL, interleukin; IFN, interferon. ELISA, enzyme linked immunosorbent assay.

Protein levels of IL-10 and interferon-gamma cytokines did not significantly differ between LC–COPD and LC in either tumor or non-tumor lung specimens (Figure 5A,B).

#### 3.4.2. Differences between Tumor and Non-Tumor Parenchyma in LC–COPD and LC Patients.

Levels of IL-10 significantly increased in the tumors compared to non-tumor specimens only in LC–COPD patients, whereas no significant differences were found in interferon-gamma levels between tumor and non-tumor lung specimens in either LC or LC–COPD patients (Figure 5A,B). A subanalysis conducted only in patients with lung adenocarcinoma revealed identical results for this set of experiments 

## 4. Discussion

In the current investigation, the number of Treg cells was greater in lung tumors of both groups of patients compared to non-tumor lung specimens. The presence of underlying COPD did not significantly modify Treg counts in the tumors. Treg cells modulate the immune system and maintain immune tolerance and homeostasis, thus preventing the development of autoimmune diseases. In general, the immunosuppressive function of Treg cells is based on the inhibition of proliferation of effector T cells [44]. In the study, Treg cells were most likely responsible for the creation of an immunosuppressive environment within the tumors; the rise in Treg cell counts was detected in a similar fashion in the tumors of both groups of patients.

Interestingly, the cytokine TGF-beta, which was shown to be significantly produced by cancer cells [7], also induces the proliferation and differentiation of Treg cells [45]. IL-10 can also be synthesized by Treg cells, which may favor the production of this cytokine in tumors, even by other cell types [34]. In the present study, a significant rise in IL-10 protein levels was detected in the tumors of the patients with underlying COPD, but not in those without this condition. These findings suggest that COPD patients are probably more prone to favor the expansion and proliferation of Treg cells within lung tumors. Future investigations should aim to explore the precise role of IL-10 and its potential relationships in lung tumorigenesis in patients with chronic airway obstruction, as in COPD. This would enable us to tease out whether the rise in IL-10 plays a significant role or may just be an epiphenomenon.

Levels of interferon-gamma did not differ between tumor and non-tumor specimens in any of the study groups. However, it has been suggested that interferon-gamma may be a potential useful biomarker for the monitoring of the response to immunotherapy [46]. Differences in clinical staging may account for the discrepancies in levels of interferon-gamma detected in the tumors of the patients in the current study and those in which high levels of this cytokine were seen in tumors of patients with advanced LC staging [46].

NK cells represent 10% of peripheral lymphocytes in patients. They are abundantly expressed in several immune structures, such as bone marrow, spleen, and lymph nodes, and the release of chemoattractants favor their migration to inflammation sites [26,27]. Importantly, NK cells stimulate maturation of dendritic cells and are also relevant for the activation of monocytes and cytotoxic T cells [28]. In the present study, the number of NK cells in tumor specimens did not differ between the two study groups of patients. Moreover, no differences were detected between lung tumor samples and non-tumor lung specimens in any of the study groups. These findings are somehow counter to previous results [47] in which NK cell infiltration degree correlated with overall survival in patients with LC. Furthermore, the tumor microenvironment was also shown to impair NK cell function, characterized by a significant reduction in their tumoricidal capacity [48].

High proportions of IgG and low proportions of IgA were associated with improved overall survival in patients with lung adenocarcinoma with specific mutations [35]. In other cancer types, high IgG proportions within the tumor lesions correlated with better survival rates among the patients [49]. A recent investigation has also demonstrated that the baseline level of anti-BP180 IgG in patients with LC was associated with a better response to immunotherapy and overall survival [50]. Furthermore, the ratio of IgA/IgG was shown to be useful as a biomarker for the early diagnosis of LC [51]. In other studies, however, IgA levels within tumors were not associated with survival in patients with hepatocellular carcinoma [52] or bladder cancer [53]. In the current study, levels of IgG-secreting plasma cells were significantly reduced within the tumor specimens only in LC patients with underlying COPD, but not in LC-only patients. Interestingly, levels of IgA did not differ between tumor and non-tumor specimens in any study group of patients. Altogether, these findings imply that the protective role of IgG was probably blunted in the tumors of the patients in the current investigation. Future studies should focus on whether IgG therapy may be effective for the treatment of lung tumors, specifically in patients with COPD. 

Finally, we would like to comment on the fact that other complementary approaches, such as flow cytometry on fresh samples, might also be used in future investigations, with the aim to identify other immune cell types within the lung tumors in COPD patients. Nonetheless, the use of relatively large fresh samples, which are required for flow cytometry, may not always be possible in these types of studies conducted on patients. 

## 5. Conclusions

The proportions of Treg cells increased in tumors of LC patients with and without COPD, while levels of IgG-secreting plasma cells decreased only in the tumors of LC–COPD patients. Protein levels of IL-10 significantly increased in tumors of LC–COPD but not in those without this condition. Levels of tumor NK cells, IgA-secreting plasma cells, or interferon-gamma did not differ between the two study groups. Immune cell subtypes and cytokines are differentially expressed in lung tumors, and the presence of underlying COPD elicited a significant decline in IgG-secreting plasma cell levels but not in the other cell types.

## Figures and Tables

**Table 1:** Clinical and functional characteristics of the study patients.

Anthropometric Variables	Lung Cancer (*n* = 20)	Lung Cancer-COPD (*n* = 33)
Age, years	65 (14)	67 (8)
Male, *n*/Female, N	10/10	29/4 **
BMI, kg/m^2^	28 (4)	25 (4) *
**Smoking History**		
Current: *n*, %	8, 40	23, 70 *
Ex-smoker: *n*, %	3, 15	9, 27
Never smoker: *n*, %	9, 45	1, 3 ***
Pack-years	18 (22)	56 (27) ***
**Lung Function Parameters**		
FEV_1_, %	88 (9)	68 (15) ***
FEV_1_/FVC, %	77 (5)	63 (8) ***
DL_CO_, %	87 (15)	72 (20) **
KCO, %	89 (13)	73 (18) **
**GOLD Stage**		
GOLD Stage I: *n*, %	NA	10, 30
GOLD Stage II: *n*, %	NA	20, 60
GOLD Stage III: *n*, %	NA	3, 10
**TNM Staging**		
Stage 0+ II: *n*, %	17, 85	28, 84.8
Stage III: *n*, %	3, 15	3, 9.1
Stage IV: *n*, %	0, 0	2, 6.1
**Histological Diagnosis**		
Squamous cell carcinoma: *n*, %	4, 20	7, 21
Adenocarcinoma: *n*, %	15, 75	25, 76
Others: *n*, %	1, 5	1, 3
**Blood Parameters**		
Total leucocytes/μL	6.39 (1.77) × 10^3^	9.52 (2.70) × 10^3^ ***
Total neutrophils/μL	3.72 (1.37) × 10^3^	6.64 (2.42) × 10^3^ ***
Total lymphocytes/μL	1.97 (0.71) × 10^3^	2.02 (0.76) × 10^3^
Albumin (g/dL)	4.4 (0.2)	4.0 (0.6) **
Total proteins (g/dL)	7.0 (0.4)	6.8 (1.0)
Fibrinogen (mg/dL)	443 (126)	427 (83)
CRP (mg/dL)	3.5 (5.6)	10.5 (19.5)
GSV (mm/h)	23 (10)	26 (16)
**Body Weight Loss, kg**		
0, *n*, %	20, 100	30, 91
1–5, *n*, %	0, 0	1, 3
6–10, *n*, %	0, 0	2, 6

Continuous variables are presented as mean and standard deviation, while categorical variables are presented as the number of patients in each group and the percentage in the study group with respect to the total population. Definition of abbreviations: N, number; kg, kilograms; m, meters; BMI, body mass index; FEV_1_, forced expiratory volume in one second; FVC, forced vital capacity; DL_CO_, carbon monoxide transfer; K_CO_, Krogh transfer factor; GOLD: Global initiative for chronic Obstructive Lung Disease; NA, not applicable; TNM, tumor, nodes, metastasis; CRP, C-reactive protein; GSV, globular sedimentation velocity; L, liter. Statistical analyses and significance: * *p* < 0.05, ** *p* < 0.01, *** *p* < 0.001 between lung cancer–Chronic Obstructive Pulmonary Disease (LC–COPD) patients and LC patients.
